# Whole-Genome Sequence of Bacillus pacificus CR121, a Fish Probiotic Candidate

**DOI:** 10.1128/mra.01206-22

**Published:** 2023-02-01

**Authors:** Sulav Indra Paul, Saif Uddin Khan, M. Khadiza Sarkar, M. Javed Foysal, Mohammad Abdus Salam, M. Mahbubur Rahman

**Affiliations:** a Institute of Biotechnology and Genetic Engineering, Bangabandhu Sheikh Mujibur Rahman Agricultural University, Gazipur, Bangladesh; b Department of Genetic Engineering and Biotechnology, Shahjalal University of Science and Technology, Sylhet, Bangladesh; c Department of Genetics and Fish Breeding, Bangabandhu Sheikh Mujibur Rahman Agricultural University, Gazipur, Bangladesh; University of Arizona

## Abstract

We describe a promising fish probiotic, Bacillus pacificus CR121, which was isolated from the intestine of a healthy Indian major carp species, rohu (Labeo rohita) and shown to possess *in vivo* disease prevention efficacy in *L. rohita* and Oreochromis niloticus. The genome sequence information will help us make use of the probiotic potential of this strain.

## ANNOUNCEMENT

Microbes residing in fish gastrointestinal (GI) tracts could be considered a potential source for isolation of probiotic strains (1). CR121 was isolated from the intestine of a healthy Labeo rohita specimen grown in the Padma River, Bangladesh. The intestine of the fish was cut aseptically, and 1 g homogenate of the intestinal segments was serially diluted and inoculated on de Man-Rogosa-Sharpe (MRS) agar plates following incubation at 28°C for 48 h. After incubation, bacterial colonies were picked from the plates. Cell-free culture supernatant of the gut bacterial isolates was screened for *in vitro* antagonistic activities, where CR121 strongly inhibited highly virulent fish-pathogenic Aeromonas veronii and Enterococcus faecalis strains (2). In *in vivo* studies, CR121 suppressed motile *Aeromonas* septicemia (MAS) in *L. rohita* (2) and Oreochromis niloticus (3), as well as streptococcosis in *O. niloticus* (3). We obtained ethics approval for the animal experiments from the Institute of Biotechnology and Genetic Engineering (IBGE) ethical review committee (approval number IBGE-ERC-008).

High-quality genomic DNA of CR121 was extracted from the original stock as used for an *in vitro* antagonistic study. A single colony was inoculated on MRS broth and incubated at 28°C for 48 h. The genomic DNA was extracted using a GeneJET genomic DNA purification kit (Thermo Fisher Scientific, USA). The quality and quantity of the extracted DNA were determined using a NanoDrop spectrophotometer (Thermo Fisher Scientific) and 0.8% agarose gel electrophoresis. Using the universal primer pair 8F (5′-AGAGTTTGATCCTGGCTCAG-3′) and 1492R (5′-GGTTACCTTGTTACGACTT-3′), the bacterium was identified as Bacillus pacificus based on the 16S rRNA gene sequence homology. A paired-end DNA library was prepared using the same DNA with the Nextera XT library prep kit (Illumina, San Diego, CA, USA) (4). Genome sequencing (600 cycles) was performed using the Illumina MiSeq benchtop sequencer (5, 6), yielding a total of 6,111,950 reads with 1,159,932,322 bases. The quality of the raw reads was determined using PRINSEQ v0.20.3 (7), and Trimmomatic v.0.38 (8) was used for trimming the low-quality sequences. The high-quality reads were assembled into a draft genome using SPAdes v3.9.0 (9). QUAST v5.0.2 (10) was used for quality assessment of the assembled genome. Genome annotation was performed using the NCBI Prokaryotic Genome Annotation Pipeline (PGAP) (https://www.ncbi.nlm.nih.gov/genome/annotation_prok/) (11). Secondary metabolite biosynthetic gene clusters (SM-BGCs) were identified using antiSMASH v6.0 (12). The genome was screened for the presence of putative bacteriocin gene clusters using BAGEL4 (13). Probiotic safety-associated genes were checked using ResFinder v4.1 (14) and the Virulence Factor Database (VFDB) (15). Default parameters were used for all bioinformatics analyses except where otherwise noted.

The *de novo* assembly resulted in an estimated genome size of 4,483,835 bp, with 121 contigs (>500 bp; largest contig, 217,233 bp), 35.81% G+C content, an *N*_50_ value of 61,542 bp, an *L*_50_ value of 22, and 258.69× genome coverage. PGAP (11) revealed a total of 4,674 genes, including 4,462 coding DNA sequences (CDSs), 91 pseudogenes, 92 tRNAs, 24 rRNAs, and 5 noncoding RNA (ncRNA) genes. RAST v2.0 (16) predicted 319 subsystems and 1,631 protein-coding genes involved in the putative functional categories of a potential probiotic bacterium, such as biosynthesis of biotin, thiamine, menaquinone, riboflavin, pyridoxine, folate, lipoic acid, etc. antiSMASH v6.0 (12) was used to detect several SM-BGCs, including pacidamycins, bacillibactin, fengycin, and molybdenum cofactor. BAGEL4 (13) was used to detect sactipeptides and colicin E9 in the genome. No antibiotic resistance genes or immune evasion genes were detected in the genome using ResFinder v4.1 (14) and VFDB (15), respectively. These genomic features signify the strain’s potential for safe use as a probiotic in aquaculture.

### Data availability.

The whole-genome shotgun project of Bacillus pacificus CR121 has been deposited at GenBank under the accession number JAPDOF000000000. The associated BioProject and BioSample accession numbers are PRJNA895596 and SAMN31516913, respectively. The raw data are available in the Sequence Read Archive (SRA) under the accession number SRX18078026. The 16S rRNA gene sequence has been deposited at GenBank under the accession number OP764053.

## References

[1] Romero J, Ringø E, Merrifield DL. 2014. The gut microbiota of fish, p 75–100. *In* Merrifield DL, Ringo E (ed), Aquacture nutrition. John Wiley & Sons, Ltd., Hoboken, NJ. doi:10.1002/9781118897263.ch4.

[2] Khan SU. 2019. Identification of native probiotic bacteria to enhance growth and prevent disease in *Labeo rohita*. MS thesis. Bangabandhu Sheikh Mujibur Rahman Agricultural University, Gazipur, Bangladesh.

[3] Rahman A. 2019. Isolation and identification of indigenous fish gut bacteria and their disease suppression effects in tilapia. MS thesis. Bangabandhu Sheikh Mujibur Rahman Agricultural University, Gazipur, Bangladesh.

[4] Illumina. 2019. Nextera XT DNA library prep: reference guide. Illumina, San Diego, CA. https://support.illumina.com/content/dam/illumina-support/documents/documentation/chemistry_documentation/samplepreps_nextera/nextera-xt/nextera-xt-library-prep-reference-guide-15031942-05.pdf.

[5] Rahman MM, Paul SI, Akter T, Tay AC, Foysal MJ, Islam MT. 2020. Whole-genome sequence of *Bacillus subtilis* WS1A, a promising fish probiotic strain isolated from marine sponge of the Bay of Bengal. Microbiol Resour Announc 9:e00641-20. doi:10.1128/MRA.00641-20. 32972930 PMC7516141

[6] Paul SI, Rahman A, Rahman MM. 2022. Whole-genome sequence of *Aneurinibacillus migulanus* TP115, a potential fish probiotic isolated from *Oreochromis niloticus* gut. Microbiol Resour Announc 26:e00861-22. doi:10.1128/mra.00861-22. PMC9670894 36301117

[7] Schmieder R, Edwards R. 2011. Quality control and preprocessing of metagenomic datasets. Bioinformatics 27:863–864. doi:10.1093/bioinformatics/btr026. 21278185 PMC3051327

[8] Bolger AM, Lohse M, Usadel B. 2014. Trimmomatic: a flexible trimmer for Illumina sequence data. Bioinformatics 30:2114–2120. doi:10.1093/bioinformatics/btu170. 24695404 PMC4103590

[9] Bankevich A, Nurk S, Antipov D, Gurevich AA, Dvorkin M, Kulikov AS, Lesin VM, Nikolenko SI, Pham S, Prjibelski AD, Pyshkin AV, Sirotkin AV, Vyahhi N, Tesler G, Alekseyev MA, Pevzner PA. 2012. SPAdes: a new genome assembly algorithm and its applications to single-cell sequencing. J Comput Biol 19:455–477. doi:10.1089/cmb.2012.0021. 22506599 PMC3342519

[10] Gurevich A, Saveliev V, Vyahhi N, Tesler G. 2013. QUAST: quality assessment tool for genome assemblies. Bioinformatics 29:1072–1075. doi:10.1093/bioinformatics/btt086. 23422339 PMC3624806

[11] Tatusova T, DiCuccio M, Badretdin A, Chetvernin V, Nawrocki EP, Zaslavsky L, Lomsadze A, Pruitt KD, Borodovsky M, Ostell J. 2016. NCBI Prokaryotic Genome Annotation Pipeline. Nucleic Acids Res 44:6614–6624. doi:10.1093/nar/gkw569. 27342282 PMC5001611

[12] Blin K, Wolf T, Chevrette MG, Lu X, Schwalen CJ, Kautsar SA, Suarez Duran HG, de los Santos ELC, Kim HU, Nave M, Dickschat JS, Mitchell DA, Shelest E, Breitling R, Takano E, Lee SY, Weber T, Medema MH. 2017. antiSMASH 4.0—improvements in chemistry prediction and gene cluster boundary identification. Nucleic Acids Res 45:W36–W41. doi:10.1093/nar/gkx319. 28460038 PMC5570095

[13] van Heel AJ, de Jong A, Song C, Viel JH, Kok J, Kuipers OP. 2018. BAGEL4: a user-friendly Web server to thoroughly mine RiPPs and bacteriocins. Nucleic Acids Res 46:W278–W281. doi:10.1093/nar/gky383. 29788290 PMC6030817

[14] Bortolaia V, Kaas RS, Ruppe E, Roberts MC, Schwarz S, Cattoir V, Philippon A, Allesoe RL, Rebelo AR, Florensa AF, Fagelhauer L, Chakraborty T, Neumann B, Werner G, Bender JK, Stingl K, Nguyen M, Coppens J, Xavier BB, Malhotra-Kumar S, Westh H, Pinholt M, Anjum MF, Duggett NA, Kempf I, Nykäsenoja S, Olkkola S, Wieczorek K, Amaro A, Clemente L, Mossong J, Losch S, Ragimbeau C, Lund O, Aarestrup FM. 2020. ResFinder 4.0 for predictions of phenotypes from genotypes. J Antimicrob Chemother 75:3491–3500. doi:10.1093/jac/dkaa345. 32780112 PMC7662176

[15] Liu B, Zheng D, Jin Q, Chen L, Yang J. 2019. VFDB 2019: a comparative pathogenomic platform with an interactive Web interface. Nucleic Acids Res 47:D687–D692. doi:10.1093/nar/gky1080. 30395255 PMC6324032

[16] Overbeek R, Olson R, Pusch GD, Olsen GJ, Davis JJ, Disz T, Edwards RA, Gerdes S, Parrello B, Shukla M, Vonstein V, Wattam AR, Xia F, Stevens R. 2014. The SEED and the Rapid Annotation of microbial genomes using Subsystems Technology (RAST). Nucleic Acids Res 42:D206–D214. doi:10.1093/nar/gkt1226. 24293654 PMC3965101

